# Soll die asymptomatische Bakteriurie bei Immunsupprimierten antibiotisch therapiert werden? Ein Rapid Review

**DOI:** 10.1007/s00120-023-02059-8

**Published:** 2023-03-20

**Authors:** Fabian P. Stangl, Julia Godly, Jennifer Kranz, Thomas Neumann, Laila Schneidewind

**Affiliations:** 1 Universitätsklinik für Urologie, Inselspital Bern, Bern, Schweiz; 2 Klinik für Urologie und Kinderurologie, Uniklinik RWTH Aachen, Aachen, Deutschland; 3 Universitätsklinik und Poliklinik für Urologie, Universitätsklinikum Halle (Saale), Halle (Saale), Deutschland; 4 Klinik für Innere Medizin C, Universitätsmedizin Greifswald, Greifswald, Deutschland; 5 Urologische Klinik und Poliklinik, Universitätsmedizin Rostock, Schillingallee 35, 18055 Rostock, Deutschland

**Keywords:** Infektiologie, Asymptomatische Bakteriurie, Antimicrobial Stewardship, Immunsuppression, Transplantation, Infections in urology, Asymptomatic bacteriuria, Antimicrobial stewardship, Immunosuppression, Transplantation

## Abstract

**Hintergrund:**

Antimikrobielle Resistenzentwicklung (AMR) stellt selbst in Europa ein essentielles Problem dar. Dies gilt insbesondere für multiresistente Escherichia-coli-Stämme, daher sollte inadäquater Antibiotikaeinsatz, insbesondere bei der asymptomatischen Bakteriurie (ASB), vermieden werden.

**Fragestellung:**

Soll die ASB bei immunsupprimierten Patienten, namentlich in der soliden Organtransplantation und insbesondere der Nierentransplantation sowie in der Stammzelltransplantation, antibiotisch therapiert werden?

**Material und Methoden:**

Es wurde eine Evidenzanalyse mittels Literaturrecherche in MEDLINE im Zeitraum 1980 bis 2022 durchgeführt. Für die Evidenzsynthese wurden lediglich RCT („randomized controlled trials“) sowie Quasi-RCT berücksichtigt.

**Ergebnisse:**

Für den Suchbegriff solide Organtransplantation und die Stammzelltransplantation konnten keine Studien identifiziert werden. Hinsichtlich der Nierentransplantation wurden drei RCT (antibiotische Therapie vs. keine Therapie) mit adulten Patienten eingeschlossen. Keine Studie zeigte einen Benefit für die antibiotische Therapie der ASB zur Vermeidung von Harnwegsinfektionen, insbesondere in der späten Transplantationsphase 2 Monate nach Transplantation. Allerdings kann die Therapie zur AMR-Entwicklung beitragen. Zusätzlich gibt es zahlreiche Evidenzlücken, z. B. bzgl. der pädiatrischen Transplantation oder zum Einfluss der Art der Immunsuppression.

**Schlussfolgerung:**

Es gibt keine Evidenz für die antibiotische Therapie der ASB in der adulten Nierentransplantation 2 Monate nach Transplantation. Doch weitere Studien hinsichtlich der aufgedeckten Evidenzlücken sind essentiell zur Vermeidung der weiteren AMR-Entwicklung.

## Abstract (en)

**Background:**

Antimicrobial resistance (AMR), especially multidrug resistant Escherichia *coli* strains, is a problem even in Europe. That is why inadequate usage of antibiotic therapy should be avoided, especially in the treatment of asymptomatic bacteriuria (ASB).

**Objectives:**

Should ASB be treated with antibiotics in immunocompromized patients, namely solid organ transplant, especially kidney transplant or stem cell transplant recipients?

**Materials and methods:**

A rapid review based on a systematic literature search in MEDLINE between 1980 and 2022 was performed. For evidence synthesis, only randomized controlled trials (RCTs) or quasi-RCTs were considered.

**Results:**

No studies were identified for the search term solid organ and stem cell transplantation. Three RCTs (antibiotic therapy versus no therapy) were included for adult kidney transplantation. None of the studies showed a benefit for antibiotic therapy of ASB in reduction of symptomatic urinary tract infections, especially in the late transplantation phase two months after kidney transplantation; furthermore, this therapy may promote AMR development. In addition, there are numerous gaps of evidence, e.g., in pediatric transplantation or regarding the influence of special immunosuppressants.

**Conclusion:**

There is no evidence for antibiotic therapy of ASB in adult kidney transplantation two months after the surgery. Further studies addressing the identified evidence gaps are essential for the prevention of further AMR development.

## Hintergrund und Fragestellung

Die antimikrobielle Resistenzentwicklung (AMR) ist eine der entscheidendsten Bedrohungen für das moderne Gesundheitswesen. Erst kürzlich veröffentlichten die European Antimicrobial Resistance Collaborators ihren Bericht zum Ausmaß der AMR in der WHO Europa Region 2019, und es ist nicht übertrieben, die Ergebnisse dieser systematischen Analyse erschreckend zu nennen. Die Autoren schätzten, dass es in der europäischen WHO-Region im Jahr 2019 541.000 Todesfälle (95 %-Unsicherheitsintervall: 370.000–763.000) mit bakterieller AMR assoziiert waren. Dabei sind sieben Erreger für die Mehrzahl der Todesfälle (*n* = 457.000) verantwortlich. Interessanterweise steht Escherichia coli dabei an erster Stelle und Aminopencillin-resistente Escherichia-coli-Stämme sind in 47 Ländern dieser Region führend für diese mit AMR assoziierten Todesfälle verantwortlich. Konsequenterweise wurde aus den Daten geschlussfolgert, dass die AMR auch in Europa eine schwerwiegende Bedrohung für die öffentliche Gesundheit darstellt und daher dringend Strategien zu deren Bekämpfung entwickelt werden müssen sowie die Unterstützung der infektiologischen Forschung gewährleistet werden muss [1].

Vor diesem Hintergrund werden Antimicrobial-Stewardship-Programme (ABS) und der differenzierte Einsatz von Antibiotika immer essentieller. Antibiotika sollten ausschließlich eingesetzt werden, wenn diese indiziert sind. Immunsuppression bzw. immunsuppressive Medikation kann das Risiko für das Auftreten insbesondere bakterieller Infektionen erhöhen [2]. Daher stellt sich die Frage, ob die asymptomatische Bakteriurie (ASB) bei diesem speziellen Patientenklientel antibiotisch therapiert werden soll. Speziell in der Nierentransplantation wird diese Frage seit Jahren kontrovers diskutiert [2–6]. Interessanterweise sind Escherichia-coli-Stämme die am häufigsten nachgewiesenen Bakterien bei Nierentransplantierten mit ASB [2–4]. Doch auch andere Patienten mit Immunsuppression sind betroffen, wie Empfänger anderer solider Organtransplantationen, z. B. Lunge oder Leber, oder Patienten nach allogener Stammzelltransplantation.

Aufgrund der ernsten Lage bzgl. der AMR-Entwicklung führten wir daher ein Rapid Review zur antibiotischen Therapie der ASB bei solider Organtransplantation (insbesondere Nierentransplantation) und allogener Stammzelltransplantation durch, um die Evidenz zusammenzufassen und Evidenzlücken für die weitere stringente Studienplanung zu identifizieren. Ein besonderer Fokus sollte dabei auf der Nierentransplantation liegen, da die Vermeidung von Transplantatpyelonephritiden für die Transplantatfunktion von erheblicher Bedeutung ist.

## Methodik

Es wurde eine schnelle Evidenzanalyse mit Literaturrecherche in MEDLINE via PubMed für den Zeitraum Januar 1980 bis Dezember 2022 durchgeführt [7]. Als Suchbegriffe wurden die Begriffe „solid organ transplantation“, „kidney transplantation“, „stem cell transplantation“ und „asymptomatic bacteriuria“ verwendet. Für die Evidenzsynthese wurden lediglich kontrollierte randomisierte Studien (RCT) und Quasi-RCT genutzt. Bezüglich der Sprache wurden nur englische und deutsche Arbeiten berücksichtigt. Der primäre Endpunkt dieser Arbeit ist die antibiotische Therapie vs. keine Therapie von ASB zur Vermeidung von Harnwegsinfektionen (HWI). Weiterhin wurden die PRISMA-Leitlinien zur Berichterstattung systematischer Übersichtsarbeiten angewandt [8].

## Ergebnisse

**Figure 1:**
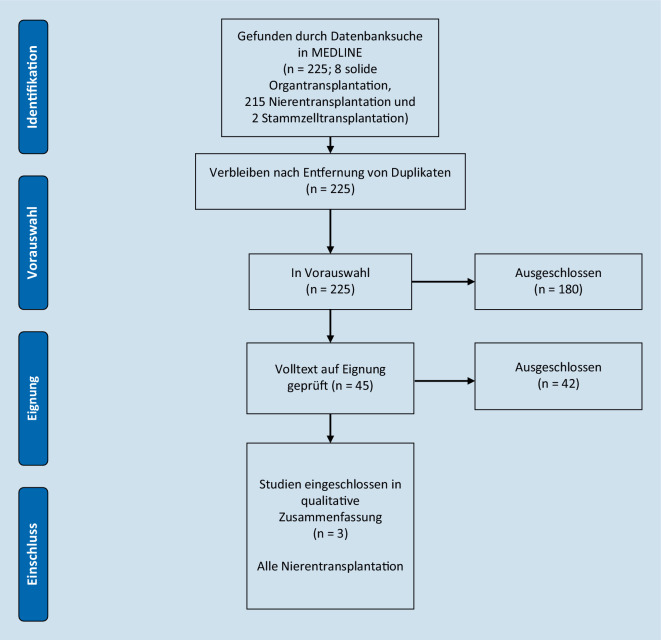

Die primäre Literatursuche ergab 8 Treffer für den Suchbegriff „solide Organtransplantation“, 215 Treffer für die „Nierentransplantation“ und 2 für die „allogene Stammzelltransplantation“, schließlich konnten nur 2 RCT sowie ein Quasi-RCT für die Nierentransplantation eingeschlossen werden (Abb. 1).

### Solide Organtransplantation

Es konnten keine RCT bzgl. unserer Fragestellung für den Suchbegriff „solide Organtransplantation“ (ausgenommen der Niere, da für diese Übersichtsarbeit im Fokus) identifiziert werden. Den aktuellsten Evidenzüberblick hinsichtlich der Thematik der ASB bieten die Leitlinien der American Society of Transplantation Infectious Diseases Community of Practice aus dem Jahr 2019 [5].

### Nierentransplantation

Unter dieser Rubrik konnten 3 Studien identifiziert werden [2–4]. Tab. 1 gibt einen ausführlichen Überblick über die Charakteristika dieser inkludierten Arbeiten. Im Jahre 2005 wurde erstmals ein Quasi-RCT zu dieser Thematik publiziert. Es konnte kein signifikanter Unterschied zwischen den Gruppen (antibiotische Therapie vs. keine antibiotische Therapie) hinsichtlich des Auftretens von HWI detektiert werden (*p* > 0,05). Zusätzlich gab es auch keinen signifikanten Unterschied bzgl. des Plasmakreatininspiegels zwischen diesen beiden Gruppen (*p* > 0,05). Daraus schlussfolgerten die Autoren, dass eine Therapie der ASB bei Nierentransplantierten nicht notwendig sei. Der erste RCT wurde dann 2016 publiziert. Hier wurde ebenfalls kein signifikanter Unterschied zwischen den Gruppen hinsichtlich des Auftretens von HWI festgestellt. Aus ihren Ergebnissen folgerten die Autoren, dass Screening und Therapie der ASB nach dem 2. Monat nach der Transplantation keinen zusätzlichen Nutzen bringt [3]. Interessanterweise gibt es einen Cochrane Review mit Metaanalyse aus diesen beiden Arbeiten [6]. Erst kürzlich, im Jahr 2021, wurden dann die Ergebnisse der BiRT-Studie publiziert. Hier wurde ebenfalls kein signifikanter Unterschied in der Inzidenz von HWI zwischen beiden Gruppen gefunden (*p* = 0,49). Außerdem war es in der Therapiegruppe wahrscheinlich, dass die zweite Episode der ASB mit resistenten Bakterien gegen gängige Antibiotika (Ciprofloxacin, Cotrimoxazol oder Drittgeneration-Cephalosporin) auftrat (*p* = 0,003). Aus diesen Ergebnissen schlussfolgerten die Autoren, dass das Screening und die nachfolgende Therapie der ASB das Auftreten von HWI bei erwachsenen Nierentransplantierten ab 2 Monaten nach der Transplantation nicht verringern kann. Allerdings erhöht die Therapie den Antibiotikaverbrauch und führt zu AMR [4].

**Table 1:** 

Referenz	Studiendesign	Hauptergebnisse	Schlussfolgerung der Autoren
*Moradi et al. 2005;* [2]	Quasi-RCT, unizentrisch, Zeitraum unklar, 88 adulte Patienten (43 in Therapiegruppe und 45 in der Kontrollgruppe), primärer Endpunkt: Inzidenz symptomatischer HWI, Follow-up: 12 Monate	Kein signifikanter Unterschied hinsichtlich des Auftretens HWI in zwischen beiden Gruppen (*p* > 0,05); kein signifikanter Unterschied hinsichtlich des Plasmakreatininspiegels zwischen den beiden Gruppen (*p* > 0,05)	Die Therapie der ASB bei Nierentransplantierten scheint die Rate der symptomatischen HWI nicht zu verringern; kurzfristig scheint die ASB die Nierenfunktion nicht zu beeinträchtigen, so dass die antibiotische Therapie hier verlassen werden kann, aber ein engmaschiges Follow-up ist notwendig
*Origüen et al. 2016;* [3]	RCT, unizentrisch, Zeitraum: Januar 2011 bis Dezember 2013, 112 adulte Patienten (53 in Therapiegruppe und 59 in der Kontrollgruppe), primärer Endpunkt: Inzidenz akuter Transplantatpyelonephritis, Follow-up: 24 Monate	Kein signifikanter Unterschied zwischen den Gruppen hinsichtlich des primären Endpunktes; keine signifikanten Unterschiede bzgl. der sekundären Endpunkte (HWI, akute Abstoßungsreaktion, Clostridium difficile Infektion, Kolonisation oder Infektion mit multiresistenten Bakterien, Transplantatfunktion und Gesamtmortalität)	Systematisches Screening und Therapie der ASB nach dem 2. Monat nach der Nierentransplantation erbringt keinen offensichtlichen Benefit für die Patienten
*Coussement et al. 2021* [4]	RCT, multizentrisch, multinational (Frankreich mit 7 Studienzentren und Belgien mit 6 Studienzentren), Zeitraum: unklar, 199 adulte Patienten (100 in der Therapiegruppe und 99 in der Kontrollgruppe), primärer Endpunkt: Inzidenz symptomatischer HWI, Follow-up: 1 Jahr	Kein signifikanter Unterschied zwischen den Gruppen hinsichtlich der Inzidenz von HWI (*p* = 0,49); 5fach höherer Antibiotikaverbrauch in der Therapiegruppe; im Vergleich zum Beginn war die zweite Episode von ASB in der Therapiegruppe signifikant häufiger mit resistenten Bakterien auf gängige Antibiotika (Ciprofloxacin, Cotrimoxazol, 3. Generation Cephalosporin) assoziiert (*p* = 0,003)	Die Screening- und Therapiestrategie für ASB kann HWI bei adulten Nierentransplantierten 2 Monate nach der Transplantation nicht verringern; allerdings begünstig diese Strategie die AMR

*RCT* randomisierte kontrollierte Studie,* HWI* Harnwegsinfektion,* ASB* asymptomatische Bakteriurie, *AMR* antimikrobielle Resistenzentwicklung

### Stammzelltransplantation

Es konnten keine RCT bzgl. unserer Fragestellung in der allogenen Stammzelltransplantation identifiziert werden. Insgesamt wurden in der primären Literatursuche auch nur 2 Publikationen gefunden: eine retrospektive Arbeit zur antibiotischen Prophylaxe in der Neutropenie und ein narratives Review über die fünf größten Herausforderungen der ABS in der Transplantationsmedizin, das es zu diskutieren gilt [9, 10].

## Diskussion

Aufgrund der dramatischen AMR-Entwicklung selbst in Europa führten wir ein Rapid Review zur Evidenzlage der antibiotischen Therapie der ASB bei immunsupprimierten Patienten durch, um inadäquaten Antibiotikaeinsatz zu vermeiden und Evidenzlücken für zukünftige Forschungsprojekte zu identifizieren. Exemplarisch für Immunsuppression wählten wir die solide Organtransplantation (insbesondere die Nierentransplantation) sowie die allogene Stammzelltransplantation aus.

Hinsichtlich des Suchbegriffs solide Organtransplantation konnte in diesem Rapid Review kein RCT identifiziert werden. Die aktuellste Zusammenfassung der Evidenzlage liefern die Leitlinien der American Society of Transplantation Infectious Diseases Community of Practice aus dem Jahr 2019, die sich aber ebenfalls auf die Nierentransplantation fokussieren [5]. Die Autoren weisen hier auf essentielle Probleme in den Studien hin, die sowohl für die klinische Praxis als auch für die weitere Forschung Bedeutung haben, so muss z. B. der Unterschied zwischen ASB und HWI innerhalb der Studien/Publikationen ganz klar definiert werden, auch bzgl. der Erregerzahl. Weiterhin sollte insbesondere im klinischen Setting an nicht-antibiotische Präventionsmethoden gedacht werden, wie z. B. die Patientenedukation hinsichtlich des Trinkverhaltens. Hervorzuheben ist außerdem der Aufruf der Autoren zur Forschung: Multizentrische retrospektive und prospektive Studien sollten standarisierte Definitionen, insbesondere zur Unterscheidung von ASB und HWI, verwenden, um Unklarheiten zu vermeiden und sich ebenfalls auf die Transplantatfunktionen in der frühen als auch der späten Phase nach der Organtransplantation fokussieren [5]. Zusammenfassend empfehlen die Autoren keine routinemäßige Therapie der ASB bei transplantierten Patienten. Allerdings kann bei zwei aufeinander folgenden positiven Urinkulturen mit > 10^5^ uropathogenen Erregern in der frühen Transplantationsphase (bis 2 Monate nach der Transplantation) eine antibiotische Therapie für 5 Tage in Erwägung gezogen werden. Aber dieses Procedere bringt vielleicht keinen zusätzlichen Benefit und fördert ggf. die AMR-Entwicklung. Nach der frühen Transplantationsphase konnten robuste Studien keinen Benefit für die antibiotische Therapie der ASB zeigen. Damit hat die empirische Antibiose bei der ASB hier keine Bedeutung – es sollte zumindest die Urinkultur mit Resistenztestung abgewartet werden und dann das passende Antibiotikum mit dem engsten Spektrum ausgewählt werden. Weiterhin sollte die ASB nicht weiter therapiert werden, wenn die zweite Urinkultur negativ ist und erneut der gleiche Erreger nachgewiesen wurde. ASB mit multiresistenten Bakterien sollte nicht therapiert werden [5].

Zur Thematik der Nierentransplantation konnten 3 relevante Studien identifiziert werden [2, 3]. Interessanterweise gibt es ein Cochrane Review mit Metaanalyse, das die beiden älteren Studien zusammenfasst. Die Cochrane-Autoren konkludierten: Aktuell gibt es nur insuffiziente Evidenz, da die Qualität der Evidenz der beiden inkludierten Studien für alle Endpunkte niedrig ist, für die Therapie der ASB bei Nierentransplantierten mit Antibiotika nach der Transplantation, aber die Datenlage ist spärlich. Weitere Studien bzgl. der Routinebehandlung mit Antibiotika könnten die Praxis beeinflussen und es werden die Ergebnisse dreier laufender RCT erwartet, die evtl. helfen, die bestehenden Unklarheiten zu beseitigen [6]. Diese Cochrane-Arbeit wurde bereits im Jahr 2018 publiziert, von Bedeutung wären folglich die 3 laufenden Studien, die hier angesprochen werden. Allerdings wurden 2 Studien bisher nicht publiziert und sind laut ClinicalTrials.gov seit über 2 Jahren nicht verifiziert worden (Stand: 08. Dezember 2022). Lediglich die BiRT-Studie, die auch in unsere Arbeit inkludiert wurde, ist publiziert worden. Hier handelt es sich methodisch um eine sehr robuste Studie, die ebenfalls keinen Vorteil für antibiotische Therapie der ASB in der späten Transplantationsphase zeigen konnte. Allerdings kann diese Antibiose zur AMR-Entwicklung beitragen [4]. Einschränkend ist zu bemerken, dass all diese Studien in der adulten Nierentransplantation durchgeführt worden sind, so dass für die pädiatrische Nierentransplantation hier keine Aussage getroffen werden kann und ein erheblicher Forschungsbedarf besteht. Außerdem muss betont werden, dass es im Setting der Nierentransplantation zahlreiche Confounder gibt, die in Studien unbedingt berücksichtigt werden müssen. Zusätzlich ist insbesondere die BiRT-Studie eine Open-label-Studie, so dass über die Art der Antibiose ebenfalls keine Aussage getroffen werden kann. Dies macht die Planung von qualitativ hochwertigen und robusten Studien zusätzlich schwierig, und die Durchführung ist so wahrscheinlich ausschließlich multizentrisch sowie multinational möglich. Solche Confounder sind z. B. die Art der Induktions- und Erhaltungsimmunsuppression, Status der Blasenentleerung, Zeitpunkt der Entfernung von Harnableitungen (Biofilmbildung), Pneumocystis-jirovecii-Prophylaxe sowie die Art und Dauer der antibiotischen Therapie. Außerdem müssen zahlreiche Outcome-Parameter betrachtet werden, wie z. B. die detaillierte Transplantatfunktion inklusive chronische Abstoßungsreaktion sowie die Kollateralschäden von Antibiotika. Insgesamt handelt es sich also um ein komplexes Problem, das eine extrem gute Studienplanung voraussetzt. Diese ist aber aufgrund der aktuellen AMR-Entwicklung absolut notwendig, denn sollten diese immunsupprimierten Patienten einen HWI mit einem multiresistenten Erreger erleiden, wird hier die Therapie sowie Eradikation umso schwieriger.

Leider konnten bzgl. der allogenen Stammzelltransplantation ebenfalls keine RCT identifiziert werden. Problematisch ist, dass hier die Situation aufgrund der langen und tiefen Neutropenie in der frühen Transplantationsphase sowie der Notwendigkeit, in dieser Phase eine antibiotische und antimykotische sowie auch antivirale Prophylaxe zu verabreichen, ungemein komplexer wird. Daher ist unserer Einschätzung nach die Planung und Durchführung von robusten Studien in Kooperation von Hämatologie und Urologie anzustreben. Durch die Literatursuche konnte hier ein interessantes narratives Review zu den fünf größten Herausforderungen der ABS in der Transplantationsmedizin identifiziert werden, das kurz angesprochen werden muss. Nach den Autoren sind diese fünf Kernprobleme: die asymptomatische Bakteriurie bei Nierentransplantierten, die febrile Neutropenie bei Stammzelltransplantierten, die antimykotische Prophylaxe bei Leber- und Lungentransplantierten, Infektionen von linksventrikulären Unterstützungssystemen und Clostridium-difficile-Infektionen [10]. Urologen sollten deshalb an der Lösung bzw. Bearbeitung des ersten Kernproblems aktiv beteiligt sein, insbesondere da wir Einfluss auf die durchgeführte Therapie sowie zukünftige Forschung haben und bei diesem Patientenklientel Escherichia coli am häufigsten vorkommt, deren resistente Stämme einen erheblichen Anteil an der AMR-Mortalität in Europa haben [1, 4].

Selbstverständlich müssen auch die Limitationen dieses Rapid Review diskutiert werden. So ist, wie es in der Natur dieser Methodik liegt, zur Suche lediglich eine Datenbank verwendet worden  und außerdem konnte für zwei Fragestellungen kein RCT identifiziert werden. Weiterhin ist die Evidenzlage spärlich und teils von geringer Qualität. Allerdings ist es, unserer Meinung nach, aufgrund der aktuellen AMR-Entwicklung extrem wichtig, auf die Problematik hinzuweisen und damit eine Übertherapie der ASB zu vermeiden. Doch die wichtigsten Ergebnisse dieser Arbeit sind die Implikationen für die Forschung, die sich ergeben haben, wie die exakte Studienplanung mit stringenten klaren Definitionen sowie der Berücksichtigung von Confoundern. Außerdem gibt es einen signifikanten Mangel an Daten für die pädiatrische Transplantation, solide Organtransplantation (ausgenommen Niere) und Stammzelltransplantation.

Abschließend möchten wir betonen, dass Infektiologie in der Urologie einen erheblichen Anteil hat und Urologen zur Reduktion der AMR-Entwicklung, insbesondere der Vermeidung der Entstehung resistenter Escherichia-coli-Stämme, beitragen können, indem wir inadäquaten Antibiotikaeinsatz vermeiden und aktiv an offenen Fragestellungen forschen [1, 11].

## Fazit für die Praxis

- Die AMR-Entwicklung (antimikrobielle Resistenzentwicklung) ist ein essentielles Problem des Gesundheitssystems, auch in Europa.
- Multiresistente Escherichia-coli-Stämme tragen signifikant zur AMR-Mortalität in Europa bei.
- Inadäquater Antibiotikaeinsatz ist zu vermeiden.
- Es gibt keine Evidenz für die antibiotische Therapie der asymptomatischen Bakteriurie (ASB) in der adulten Nierentransplantation 2 Monate nach Transplantation.
- Allerdings ist Datenlage begrenzt und teils lückenhaft. Zukünftige Forschung ist insbesondere in der pädiatrischen Transplantation und Stammzelltransplantation essentiell.
- Zukünftige Studien müssen robust geplant werden und zahlreiche Confounder, wie die Art der Immunsuppression, berücksichtigen.
